# Radiological Findings on Chest Computed Tomography in Patients With the Primary Diagnosed Chronic Lymphoproliferative Diseases

**DOI:** 10.7759/cureus.22935

**Published:** 2022-03-07

**Authors:** Ganna Usenko, Kateryna Gashynova

**Affiliations:** 1 City Hematology Center, Public Non-profit Enterprise “City Clinical Hospital #4” of Dnipro City Council, Dnipro, UKR; 2 Department of Occupational Diseases, Clinical Immunology and Clinical Pharmacology, Dnipro State Medical University, Dnipro, UKR

**Keywords:** chronic lymphoproliferative diseases, lesions of the lung parenchyma, lymphadenopathy, chest ct, comorbidity

## Abstract

Introduction

The presence of concomitant respiratory pathology complicates the process of treatment and recovery of patients with chronic lymphoproliferative diseases (CLDs). Therefore, the diagnosis of lung injury is an important step in the management of such patients.

Objectives

The aim of this study was to determine the prevalence, nature, extent, and location of changes diagnosed by high-resolution chest computed tomography (CT) in patients with CLDs at the initial examination.

Methods

Medical records of inpatients who were hospitalized in 2018-2019 to the City Hematology Center of the Public Non-Profit Enterprise “City Clinical Hospital #4” of Dnipro City Council with a confirmed clinical diagnosis of CLDs were included in the retrospective study. The results of initial high-resolution chest CT were studied and analyzed.

Results

Out of 1,004 hospitalized patients with confirmed CLDs, 119 patients were primarily diagnosed. Among them, 81 patients underwent chest CT examination (68.1%) before the beginning of specific therapy. The average age was 65 (56;68) years, 46 (56.8%) were men. 23 (28.4%) patients were diagnosed with chronic lymphocytic leukemia, 28 (34.6%) patients with multiple myeloma, 24 (29.6%) patients with lymphoma, and six patients (7.4%) had other CLDs. It was found that both central and peripheral lymphadenopathy had about a third of the studied cohort of patients (33.3 and 29.6%, respectively), and these symptoms dominated in patients with chronic lymphocytic leukemia (43.5 and 50%, respectively), lymphoma (50 and 52.2%, respectively), and other CLDs (45.8 and 16.7%, respectively), in contrast to patients with multiple myeloma (7.1 and 0%, respectively). Lesions of the lung parenchyma were found in 45.7% of the studied cohort and were met more often in patients with multiple myeloma (67.9%). However, when comparing the prevalence of their categories, no statistically significant differences were found. Predictable bone-destructive changes were statistically significantly more common in patients with multiple myeloma than in other groups of patients with CLDs (*P*=0.0003).

Conclusions

Signs of pulmonary diseases during initial chest CT were found almost in half of the patients with CLDs. It potentially may affect the frequency of treatment complications in such patients. Lymphadenopathy was the most common finding on chest CT, especially in patients with chronic lymphocytic leukemia and lymphoma. And enlarged intrathoracic lymph nodes possibly could lead to pulmonary functions disorders. Among the types of lung parenchyma lesions, pneumofibrosis and foci of consolidation in the lower lung lobes were the most often diagnosed. Chest CT is informative at the stage of the initial examination of patients with CLDs not only for clinical diagnosis but also for the diagnosis of respiratory comorbidities and prediction of the disease outcome and treatment complications.

## Introduction

Chronic lymphoproliferative diseases (CLDs) are pathological conditions of heterogeneous nature, the factors of development of which are considered to be both environmental factors and genetic pathology [1]. The most common CLD is chronic lymphocytic leukemia (CLL) with a global incidence of up to five per 100,000 population and a higher prevalence among men [2]. Multiple myeloma (MM) ranks second among oncohematological pathologies in prevalence (about four cases per 100,000 population), which increases with age [3]. Various pathomorphological variants of lymphomas are also common among CLDs. For example, non-Hodgkin’s lymphoma occurs in 19.7 cases per 100,000 population in the United States, and its prevalence increases with age (from 9.3 per 100,000 under 65 years and up to 91.5 per 100,000 population among people over 65 years) and almost twice more common among men (23.9 per 100,000) than among women (16.4 per 100,000 population) [4].

Management of patients with CLDs is a quite difficult task because in addition to issues of effectiveness, complications, and side effects that occur with different types of treatment are extremely important for the prognosis. A common side effect of chemotherapy and radiation therapy is pulmonary toxicity, which leads to various irreversible changes in the lung parenchyma and airways and, as a consequence, to respiratory disorders [5,6]. A significant share of complications in various types of therapy for CLDs are infections, and in the first place-respiratory lesions. Such events are often fatal and determine the life expectancy of patients [7,8].

The presence of concomitant respiratory pathology complicates the process of treatment and recovery of patients with CLDs, as it may be a potential risk factor for an unfavorable prognosis for this category of patients. In addition, this can complicate the diagnostic process, for example, up to 93% of patients with sarcoidosis have such a common syndrome among patients with CLDs as intrathoracic lymphadenopathy [9]. However, this problem is not thoroughly studied today, and there are only a few works on this issue. Thus, according to a study conducted at the Mayo Clinic (USA) and published in 2017 [10], 17% of patients with CLDs had respiratory diseases, and among patients with CLL who died, 23% had respiratory comorbidity. The study previously conducted at our center showed the presence of diagnosed respiratory comorbidity in 9% of patients [11]. Simultaneously, respiratory symptoms were much more common: as an example, shortness of breath had 14.1 % of primary diagnosed with CLDs patients.

According to the Unified Clinical Protocol for the care of patients with lymphoma (2013), chest computed tomography (chest CT) at the stage of initial examination and formulation of clinical diagnosis is mandatory and is carried out to diagnose lymphadenopathy [12], however, diagnosis of lesions of the lung parenchyma and airways is not regulated by protocols. On the other hand, in clinical practice chest CT is not performed in all patients and sometimes is still replaced by chest X-ray. CT is a fundamental method of radiological diagnosis of chest diseases. Its application avoids the necessity for a significant number of invasive diagnostic techniques [13]. The sensitivity of modern CT in the diagnosis of thoracic diseases is over 94% and is the most effective in comparison with other imaging methods [14]. Because the treatment of this category of patients uses drugs that have immunosuppressive and toxic effects, it is important to monitor not only the lymph nodes but also the lung parenchyma, to diagnose infectious complications and the formation of mucositis and pneumofibrosis. Therefore, the problem of determining the nature, extent, and location of lesions of the respiratory system at the stage of initial diagnosis of CLDs is extremely relevant, but today remains open in Ukraine and the world. The study aimed to determine the prevalence, nature, extent, and location of changes diagnosed by high-resolution chest CT in patients with CLDs at the initial examination.

## Materials and methods

Medical records of inpatients who were hospitalized in 2018-2019 in the City Hematology Center of the Municipal Non-Profit Enterprise “City Clinical Hospital № 4” of Dnipro City Council with a confirmed clinical diagnosis of CLD were included in the retrospective study. The results of high-resolution chest CT, which was made during hospitalization using CT (Toshiba Aquilion 160, Japan), were studied. The presence of enlarged lymph nodes (mediastinal paraaortic, paratracheal, sub-carinal, and hilar lymph nodes; and peripheral axillary, cervical and subclavian), nodules, cysts, thickening of the walls of the bronchi, interalveolar septa, areas of ground-glass opacity, consolidation and destruction in the lung parenchyma, pleural effusion, bone-destructive changes were assessed. Statistical analysis was performed in Excel AtteStat 2010 (License number 02260-018-0000106-48794). Quantitative variables were presented as the mean (standard deviation, SD) with the normal distribution of data or as the median (interquartile range) with the abnormal distribution. The distribution of variables was analyzed using the Shapiro-Francia test [15]. Qualitative variables were represented as absolute numbers and percentages (n; %), comparisons of several binary sets were performed using the Chi-square criterion.

## Results

A total of 1,004 hospitalized patients with confirmed CLDs were included in the analysis. The average age of hospitalized patients was 65 (56; 68) years, 523 (52.1%) of them were men. The average duration of hospitalization was 9 (6; 13) days and mortality was 2.4%. Four hundred eighty-nine (48.7%) patients were diagnosed with MM, 334 (33.3%) with CLL, 136 (13.5%) with lymphoma, and 45 (4.5%) patients had other CLDs. One hundred nineteen (11.9%) case histories belonged to patients who were hospitalized for the first time after diagnosis, 885 (88.1%) to patients who had rehospitalizations. Further analysis was performed on 119 inpatient medical records for the first time diagnosed with CLDs. The vast majority of subjects were patients with MM, CLL, and lymphoma. Detailed characteristics of the study population are given in Table 1.

**Table 1:** Demographical and clinical characteristics of patients with the primary diagnosis of CLDs CLL: chronic lymphocytic leukemia; MM: multiple myeloma; CLD: chronic lymphoproliferative disease

Characteristics, units of measurement of the disease	CLL (n = 30)	ММ (n = 51)	Lymphoma (n = 27)	Other CLD (n = 11)	Р-value
The median age (25%;75%), years	66 (58;68)	65 (57;69)	64 (50;68)	65 (49;73)	0.59
Men sex, n (%)	19 (63.3)	25 (49)	10 (37)	8 (72.7)	0.11
The median duration of hospitalization (25%;75%), days	11.5 (8;18)	15 (11;20)	13 (8;18)	9 (8;18)	0.22
Mortality, n (%)	2 (6.7)	3 (5.9)	1 (3.7)	1 (9.1)	0.93
The presence of respiratory comorbid pathology, n (%)	2 (6.7)	9 (17.6)	5 (18.5)	0 (0)	0.24

Groups of patients with different CLDs did not differ statistically significantly in age, sex, duration of hospitalization, mortality, and the presence of diagnosed respiratory comorbidity (p>0.05 by Kruskal-Wallis test). Among patients with CLL, respiratory comorbidity was found in two patients-community-acquired pneumonia in one patient (0.8% of the entire cohort and 3.3% of patients with CLL) and chronic bronchitis in one patient (0.8% of the total cohort and 3.3% of patients with CLL); among patients with lymphoma respiratory pathology was registered in five patients-chronic obstructive pulmonary disease (COPD) in one patient (0.8% of the whole cohort and 3.7% of patients with lymphoma), asthma in one patient (0.8% of the whole cohort and 3.7% of patients with lymphoma), sinusitis in one patient (0.8% of the entire cohort and 3.7% of patients with lymphoma), and mucositis in two patients (1.7% of the entire cohort and 7.4% of patients with lymphoma); among nine patients with MM and respiratory comorbidity, one patient had sinusitis (0.8% of the whole cohort and 1.9% of patients with MM), one patient had tracheitis (0.8% of the whole cohort and 1.9% of patients with MM), two patients with chronic bronchitis (1.7% of the entire cohort and 3.9% of patients with MM), and community-acquired pneumonia in five patients (4.2% of the total cohort and 9.8% of patients with MM). Patients with chronic respiratory comorbidity had a stable phase when were enrolled in the study. Reported community-acquired pneumonia in six cases was present at the time of initial diagnosis of CLDs. Out of 119 patients, 81 patients underwent chest CT examination (68.1%), while chest radiography in two projections was performed in 61 patients (51.3%) at the primary care level. Both types of studies were performed in 23 patients (19.3%).

Among the primary patients who underwent chest CT (n = 81), the average age was 65 (56; 68) years, 46 (56.8%) were men. Twenty-three (28.4%) patients were diagnosed with CLL, 28 (34.6%) patients with MM, 24 (29.6%) patients with lymphoma, and six patients (7.4%) had other CLDs (Waldenstrom’s macroglobulinemia, plasmacytoma, hairy cell leukemia). Respiratory comorbidity was identified in 11 cases (13.6%): five patients (6.2%) had the diagnosis of community-acquired pneumonia, three patients (3.7%) had chronic bronchitis, one patient (1.2%) had COPD, one patient (1.2%) had asthma, and one patient (1.2%) had a diagnosis of mucositis.

During analyzing the nature of changes in chest CT in patients with newly diagnosed CLDs (Table 2), it was found that about a third of the studied cohort of patients had both central and peripheral lymphadenopathy. These symptoms dominated in patients with CLL, lymphoma, and other CLDs, in contrast to patients with MM. In turn, patients with MM had parenchymal lesions statistically significantly more frequently. However, no statistically significant differences were detected when the prevalence of their categories was compared. Predictably, bone-destructive changes were statistically significantly more common in patients with MM than in other groups of CLDs patients.

**Table 2:** Characteristics of chest CT changes in patients with a newly diagnosed CLDs CLL: chronic lymphocytic leukemia; MM: multiple myeloma; CLD: chronic lymphoproliferative disease

Characteristics, units of measurement disease	Total (n = 81)	CLL (n = 23)	ММ (n = 28)	Lymphoma (n = 24)	Other CLDs (n = 6)	P-value
Central lymphadenopathy (mediastinal), n (%)	27 (33.3)	10 (43.5)	2 (7.1)	12 (50)	3 (50)	0.0004
Peripheral lymphadenopathy, n (%)	24 (29.6)	12 (52.2)	0 (0)	11 (45.8)	1 (16.7)	0.0001
Pleural effusion, n (%)	9 (11.1)	3 (13)	3 (10.7)	3 (12.5)	0 (0)	0.83
Bone-destructive changes, n (%)	11 (13.6)	0 (0)	10 (35.7)	0 (0)	1 (16.7)	0.0003
Lesions of the lung parenchyma						
All the categories, n (%)	37 (45.7)	7 (30.4)	19 (67.9)	10 (41.7)	1 (16.7)	0.02
Ground-glass opacity, n (%)	0 (0)	0 (0)	0 (0)	0 (0)	0 (0)	1.0
Thickening of interalveolar septa, n (%)	0 (0)	0 (0)	0 (0)	0 (0)	0 (0)	1.0
Thickening of the walls of the bronchi, n (%)	12 (14.8)	3 (13)	7 (25)	1 (4.2)	1 (16.7)	0.21
Consolidation, n (%)	11 (13.6)	3 (13)	6(21.4)	2 (8.4)	0 (0)	0.4
Cysts, n (%)	0 (0)	0 (0)	0 (0)	0 (0)	0 (0)	1.0
Nodules, n (%)	3 (3,7)	0 (0)	1 (3,6)	2 (8,4)	0 (0)	0.47
Cavities, n (%)	0 (0)	0 (0)	0 (0)	0 (0)	0 (0)	1.0
Pneumofibrosis, n (%)	14 (17.3)	2 (8.7)	5 (17.9)	6 (25)	1 (16.7)	0.53

**Figure 1:**
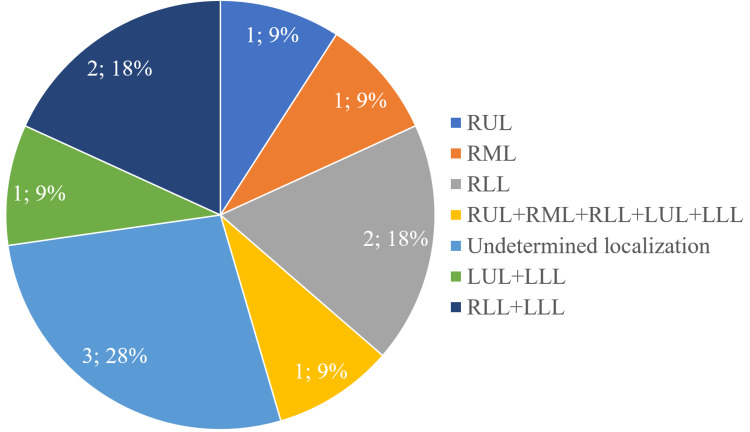
Localization of foci of consolidation in the pulmonary parenchyma in patients with CLDs RUL: right upper lobe; RML: right middle lobe; RLL: right lower lobe; LUL: left upper lobe; LLL: left lower lobe

Eleven patients (13.6%) had foci of consolidation in the pulmonary parenchyma, the localization is presented in Figure 1.

**Figure 2:**
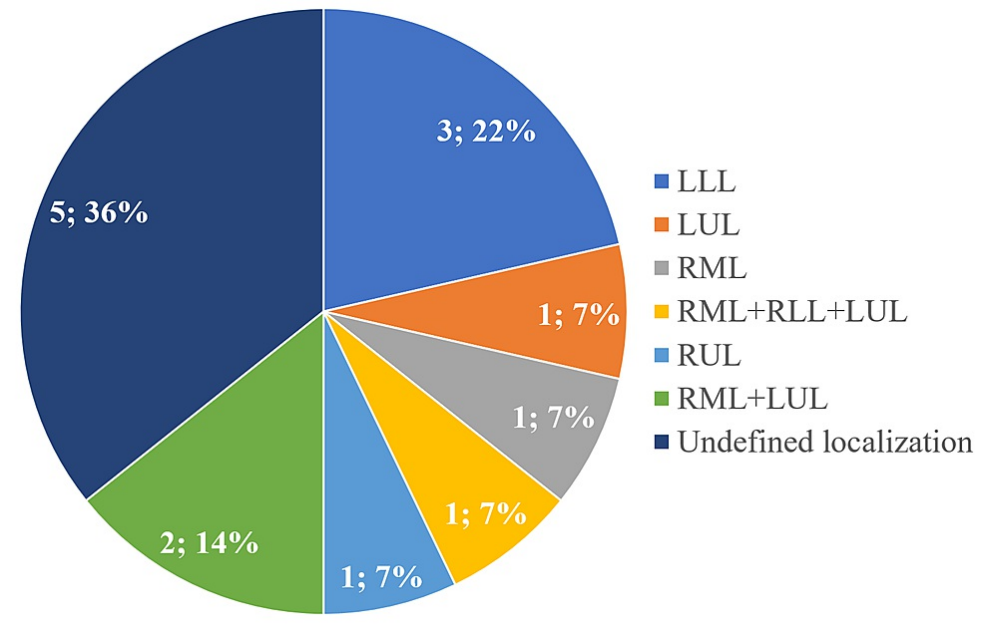
Localization of pneumofibrosis in patients with chronic lymphoproliferative disorders RUL: right upper lobe; RML: right middle lobe; RLL: right lower lobe; LUL: left upper lobe; LLL: left lower lobe

Nodules were detected in three patients (3.7%), had a diameter of up to 3.5 mm, and were localized in the right middle lobe (RML), right lower lobe (RLL), and left upper lobe (LUL). Bronchial wall thickening was determined in 12 patients among newly diagnosed patients with CLDs who underwent chest CT before the start of specific treatment (14.8%). One patient (8.3%) had bronchial wall thickening in all lung lobes, two patients (16.6%) only in LLL, one (8.3%) only in LUL, and one (8.3%) in the lower lobes of both lungs. Pneumofibrosis was determined on chest CT in 14 patients (18.3%), the localization is presented in Figure 2. Among them, one patient (7.1%) had a clinical diagnosis of chronic obstructive pulmonary disease.

Single calcifications in the lung parenchyma were found in seven patients (8.6%), were up to 3 mm in size, and localized in LUL (3; 42.9%), RML (2; 28.6%), and LLL (2; 28.6%). Multiple calcifications were detected in two patients (2.5 %) and localized in LLL in both cases. Bone-destructive changes on chest CT were detected in 11 patients with CLDs (13.6%), of which 10 patients were diagnosed with MM (90.9%) and one with another CLD-hairy cell leukemia (8.1%). Among them, eight patients had osteolytic focal changes (72.7%) with predominant localization was in the cervical and thoracic spine, ribs, sternum, and clavicle. Fractures were found in six patients (54.5%), four of them in the thoracic vertebrae (66.7%) and two in the ribs (33.3%).

**Figure 3:**
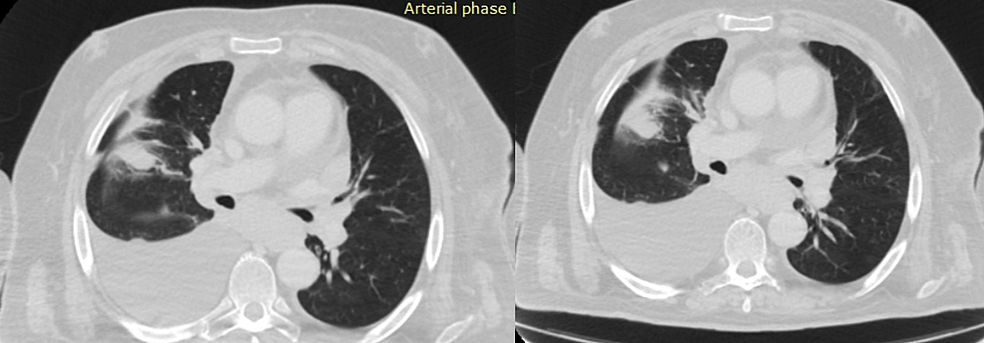
Chest CT of the patient with lymphoma (pleural effusion, pneumofibrosis, consolidation, central lymphadenopathy are presented)

**Figure 4:**
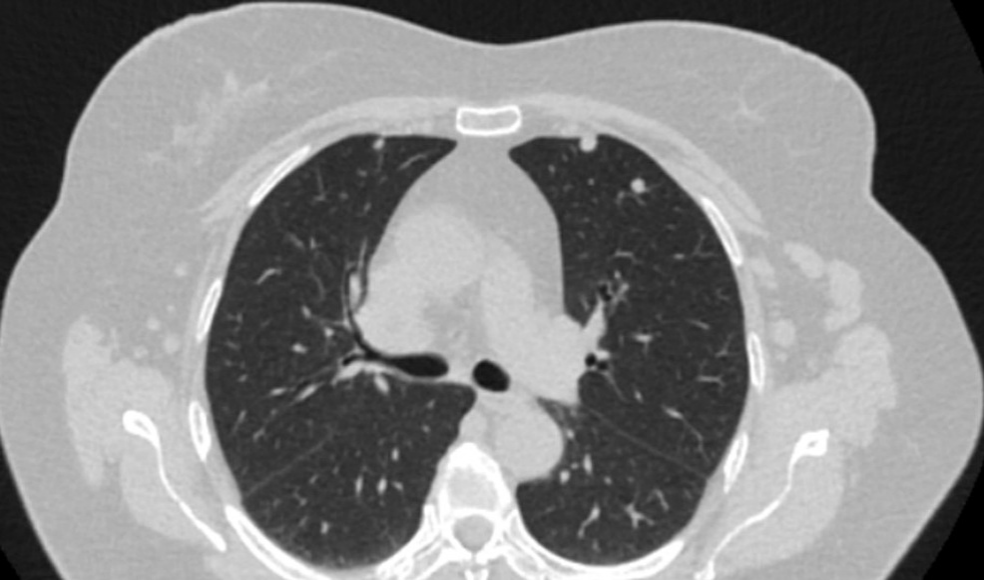
Chest CT scan of the patient with chronic lymphocytic leukemia (nodular lesion is presented)

Lymphadenopathy on chest CT was detected in 31 patients (38.3%), including 13 patients with CLL (41.9%), 13 with lymphoma (41.9%), two patients with MM (6.5%), and three with other CLDs (9.7%). Mediastinal lymph nodes predominated among the enlarged lymph nodes detected on chest CT (33.3%), including paraaortic in nine patients (11.1%), paratracheal in five (6.2%) patients, subcarinal in four (4.9%) patients, and hilar lymph nodes in 10 primary patients with CLDs (12.3%). Peripheral lymphadenopathy occurred in 24 (29.6%) cases: axillary lymph nodes were enlarged in 21 patients (25.9%), cervical lymph nodes in eight patients (9.9%), and subclavian in five patients (6.2%). Chest CT scans of the patients from the study cohort are presented in Figures 3-4.

## Discussion

The major strength of this study includes a homogeneous cohort of patients with chronic lymphoproliferative disorders. This is the first study in Ukraine reporting chest CT findings in patients with CLDs, and there are practically no world publications on this topic. Almost half of the patients with CLDs at the initial examination with chest CT revealed signs of pulmonary diseases, which should be taken into account when predicting complications of treatment for such patients. The most common pathological changes detected by chest CT in these patients were lymphadenopathy (about 40% of patients). One-third of patients with CLDs had an increase in mediastinal lymph nodes, including 6.2% of paratracheal lymph nodes, 4.9% of bifurcation, and 12.3% of hilar lymph nodes, which could potentially affect airway patency and pulmonary function. Predictable, lymphadenopathy was more common in patients with CLL and lymphoma, but also was detected in 7% of patients with MM, which is not classical for this disease and needs further diagnosing to detect the possible primary cause of lymphadenopathy in these patients. Among the lesions of the lung parenchyma, pneumofibrosis with predominant localization in the lower lobe of the left lung and foci of consolidation of the lung parenchyma in the lower lobes of both lungs were most often diagnosed.

Some limitations of our study should be acknowledged. First, the monocentric design has an impact on the generalizability of these findings, so data can be extrapolated with caution to the entire patient population. Second, the study was retrospective, so not everyone patient had a chest CT scan. At the stage of initial examination and formulation of clinical diagnosis, chest CT was performed only in 68% of patients. Finally, data on comorbidity were collected only from medical records, consequently, we did not have an opportunity to exclude the likeliness of comorbidity underdiagnosing. Future studies will have a prospective design that will allow us to assess the real prevalence of respiratory comorbidity in patients with CLDs (using physical examination, anamnesis data, chest CT in all patients, and spirometry), as well as its relationship with the outcome of the underlying disease.

## Conclusions

Data from this study indicates the need to take immediate measures to improve the use of chest CT at the stage of the initial examination of patients with CLDs not only for confirming the main diagnosis but also for the diagnosis of concomitant respiratory pathology. These possibly could predict the complication of the treatment and improve the prognosis for patients.
